# Management, prognosis and predictors of unfavourable outcomes in patients newly hospitalized for transient ischemic attack: a real-world investigation from Italy

**DOI:** 10.1186/s12883-017-0796-3

**Published:** 2017-01-19

**Authors:** Giovanni Corrao, Federico Rea, Luca Merlino, Paolo Mazzola, Federico Annoni, Giorgio Annoni

**Affiliations:** 1 Laboratory of Healthcare Research & Pharmacoepidemiology, Unit of Biostatistics, Epidemiology and Public Health, Department of Statistics and Quantitative Methods, University of Milano-Bicocca, Milan, Italy; 2 Operative Unit of Territorial Health Services, Lombardy Region, Milan, Italy; 3 Department of Medicine and Surgery, University of Milano-Bicocca, Monza, Italy; 4 Department of Pathophysiology and Transplantation, University of Milano, Milan, Italy; 5 Geriatric Unit, San Gerardo University Hospital, Monza, Italy

**Keywords:** Transient ischemic attack, Healthcare utilization database, Population-based cohort study, Mortality, Stroke, Acute myocardial infarction

## Abstract

**Background:**

Understanding the gap between evidence-based recommendations and real-world management is important to inform priority setting and health service planning.

**Methods:**

The 7,776 residents in the Italian Lombardy Region who were newly hospitalized for transient ischemic attack (TIA) during 2008–2009 entered into the cohort and were followed until 2012. Exposure to medical care including selected drugs, diagnostic procedures and laboratory tests was recorded. A composite outcome was employed taking into account all-cause death and hospitalization for stroke and acute myocardial infarction. A multivariable proportional hazards model was fitted to estimate hazard ratio, and 95% confidence intervals (CI), for the exposure-outcome association.

**Results:**

During the first year after discharge, 8.6, 49.7 and 48.5% of patients did not use any drugs, diagnostic procedures and laboratory tests respectively. Patients exposed to medical care had 59% reduced risk (95% CI, 50 to 66%) with respect to those who did not use any of these services.

**Conclusions:**

Although the Italian National Health System supplies universal coverage for healthcare, several TIA patients receive suboptimal care. Systematic improvements are necessary in order to improve patient outcomes.

## Background

Observational studies have shown that transient ischemic attack (TIA) not only predisposes to a very high early stroke risk [1, 2], but is also associated with long-term increased risk of stroke, myocardial infarction and vascular death [3–5].

Although evidence-based guidelines for acute treatment and secondary prevention for TIA have been developed along with improved diagnostic procedures and treatments [6–10], management of TIA in clinical practice is variable and often suboptimal [11–14]. Understanding the gap between evidence-based recommendations and real-world management is therefore important to inform priority setting and health service planning.

We performed a population-based study, investigating the cohort of patients discharged from hospital where they were admitted for their first episode of acute TIA. The purposes of the present study were to evaluate the patterns of practice in the management of these patients, to measure the rate of selected outcomes and to assess the impact of the observed patterns on the risk of selected outcomes.

## Methods

### Setting

The data used for this study were retrieved from the healthcare utilization databases of Lombardy, a region of Italy which accounts for almost ten million individuals (about 16% of the total population). Italian citizens are beneficiaries of the National Health Service (NHS), which provides universal free-of-charge coverage for many healthcare services. Since 1997, the NHS management in Lombardy utilizes an automated system of databases that collects a variety of information, including hospital admissions from public or private hospitals, outpatient dispensations of drugs, visits in highly specialized ambulatories and services in diagnostic rooms.

For each patient we linked the above databases using a single identification code. In order to preserve privacy, each identification code was automatically converted into an anonymous code. The inverse process was prevented by deletion of the conversion table [15]. Details of healthcare utilization databases of the Lombardy Region and their use in the field of cardiovascular (CV) diseases have been reported elsewhere [16–18]. According to the rules from the Italian Medicines Agency (available at: http://www.agenziafarmaco.gov.it/sites/default/files/det_20marzo2008.pdf), retrospective studies using administrative databases do not require Ethics Committee protocol approval.

### Study cohort

All beneficiaries of the NHS who were hospitalized at least once with a diagnosis of TIA (ICD-9^th^ codes, 433.10, 433.30, 434.00, 434.10, 434.90, 435.x) during the period 2008–2009 were identified, and the date of first hospital discharge recording this diagnosis was defined as the index date. Patients were excluded whether: (1) they already experienced any cerebrovascular hospitalization in the eight years preceding the index date; (2) they were admitted for a planned hospital access (i.e., only patients who had access to the hospital from emergency wards were included); (3) they died during the index admission. The remaining patients constituted the study cohort.

Each cohort member was followed from the index date until censoring, i.e., the earliest among the dates of outcome onset (see below), emigration, or December 31^st^, 2012.

The outcome was experienced by cohort members who during follow-up died for any cause or were hospitalized for stroke (ICD-9^th^ codes, 430–434 except 433.10, 433.30, 434.00, 434.10, 434.90) or acute myocardial infarction (AMI) (ICD-9^th^ code 410). A patient who experienced at least one of these events was considered as having the outcome. The earliest date of hospital admission for these events was considered as the time of outcome.

### Reference cohort

A reference cohort suitable to be used as comparator for the study cohort was generated. Reference cohort members were NHS beneficiaries 1:1 matched on gender and age at cohort entry with TIA cohort members, who did not yet experience hospitalization for TIA at index date and were at risk for the outcome at the time when the matched TIA patient was discharged. Each member of the reference cohort was followed for identifying those who experienced the considered outcomes exactly as described for the study cohort.

### Baseline characteristics and healthcare exposures

Gender, age, nationality (Italian born or migrant), and the Charlson comorbidity index score [19] calculated by using the diagnostic information available from inpatient charts in the five-year period prior the index date, were recorded at baseline for each study and reference cohort member. During the one-year periods before and after the date of the index hospital admission, as well as during the entire period of follow-up, we recorded the following information: (i) use of selected drugs (blood pressure- and lipid-lowering agents, antiplatelet, oral anticoagulants and antiarrhythmic drugs and antidiabetics); (ii) exposure to diagnostic procedures (i.e., carotid, transcranial and cardiac doppler ultrasonography, neuroimaging – computed tomography or magnetic resonance imaging – or cerebral angiography, and electrocardiography); and (iii) laboratory tests (i.e., total cholesterol and glycated haemoglobin).

### Data analysis

McNemar chi-square test for matching pairs was used to compare healthcare exposures experienced by (i) study and reference cohort during the one-year period before the index date, (ii) study cohort one year before and one year after the index date.

Chi-square test was used to compare the proportion of study cohort members who did not use healthcare services according to gender, age (patients younger or older 70 years), and Charlson comorbidity index score (none or at least one sign of comorbidity).

The cumulative probability of experiencing a specific outcome (i.e., death or hospital admission for stroke or AMI) during follow-up was estimated by the competing risks method [20]. With this approach a subject was assumed to experience the outcome only once, and the overall incidence at a given time was split into the sum of the cause-specific cumulative incidences.

Finally, the Cox proportional hazard regression was used to estimate the hazard ratio (HR) and 95% confidence interval (CI), for the association between healthcare exposure and time of the outcome onset (i.e., the earliest date between those of death or hospital admission for stroke or AMI). Because exposure to healthcare varies over time, assessment of its value requires proper accounting for the cumulative and varying nature of the measure. This was done by including healthcare exposure categories as time-dependent variables in the model [21]. Adjustment was made for the baseline characteristics by including the corresponding covariates into the model. For each time-fixed covariate included in a Cox proportional hazards model, the proportionality assumption was assessed graphically by survival curves (Kaplan-Meier method).

To verify the robustness of our findings, the aforementioned analysis was limited to those patients who during the index hospital admission were underwent to computed tomography and/or magnetic resonance imaging and/or cerebral angiography.

For all hypotheses tested, two-tailed *p*-values less than 0.05 were considered as significant.

## Results

Among the 19,767 patients hospitalized with a diagnosis of TIA during the period 2008–2009, 11,991 were excluded; 5,822 (48.5%) because they had previous cerebrovascular hospitalization; 6,038 (50.4%) because they were admitted for a planned hospital access; and 131 (1.1%) because they died during the index admission. The remaining 7,776 patients formed the study cohort. At baseline, their mean age was 73.3 years (SD, 13.0 years, range 18–101 years), 52.9% of them were women, 96.6% was Italian native and 89.3% of them did not have signs of comorbidities. During the index hospital admission 6,063 patients (78.0%) were underwent to at least a neuroimaging procedure, 4,808 (61.8%) to computed tomography only, 395 (5.1%) to magnetic resonance only, 828 (10.6%) to both computed tomography and magnetic resonance and 36 (0.5%) to cerebral angiography.

Table 1 shows that one year before the index hospitalization, study cohort members had significantly higher use of healthcare services than the corresponding reference cohort members, except for exposure to lipid-lowering agents. As a consequence, there was statistical evidence that prevalence of patients who did not use drugs, diagnostic procedures and/or laboratory tests was significantly higher among reference than TIA study cohort members. From one year before to one year after the index hospital admission for TIA, study cohort members increased the exposure to healthcare services. There was evidence that the number of patients who did not experience use of drugs and/or diagnostic procedures diminished after hospital admission for TIA, even if the proportion of individuals who did not undergo laboratory tests did not change during the considered time-frame. During the first year after discharge, of the 7,776 patients of the study cohort, 667 (8.6%) did not use any medication, 3,863 (49.7%) any diagnostic procedure, and 3,775 (48.5%) any laboratory test, while 388 of them (5.0%) did not show any evidence of exposure to none of the considered healthcare services. The latter were mainly represented by women and elderly, being the prevalence of healthcare no users 4.2 and 5.7% among men and women respectively (*p* = 0.0029) and 4.3 and 5.4% among patients younger and older than 70 years respectively (*p* = 0.0355). There was no evidence that prevalence of healthcare no users differed among patients with or without signs of comorbidity.

**Table 1:** Comparison of healthcare exposure of reference cohort (A) and transient ischemic attack (TIA) study cohort before (B) and after (C) index hospitalization

	Reference cohort	TIA study cohort		*p*-value	
	One year before index hospitalization (A)	One year before index hospitalization (B)	One year after index discharge (C)	(A vs. B)	(B vs. C)
Drugs					
Blood pressure-lowering agents	4,544 (58.4%)	5,255 (67.6%)	5,819 (74.8%)	<0.0001	<0.0001
Lipid-lowering agents	1,358 (17.5%)	1,446 (18.6%)	2,651 (34.1%)	0.0612	<0.0001
Antiplatelet drugs	2,162 (27.8%)	2,754 (35.4%)	5,926 (76.2%)	<0.0001	<0.0001
Oral anticoagulants	421 (5.4%)	499 (6.4%)	997 (12.8%)	0.0077	<0.0001
Antiarrhythmics	345 (4.4%)	416 (5.3%)	535 (6.9%)	0.0082	<0.0001
Antidiabetics	867 (11.1%)	1,233 (15.9%)	1,300 (16.7%)	0.0192	0.0002
None of the previous	2,720 (35.0%)	1,958 (25.2%)	667 (8.6%)	<0.0001	<0.0001
Diagnostic procedures					
Cardiac doppler ultrasonography	690 (8.9%)	845 (10.9%)	1,075 (13.8%)	<0.0001	<0.0001
Carotid doppler ultrasonography	633 (8.1%)	759 (9.8%)	1,172 (15.1%)	0.0004	<0.0001
Neuroimaging or cerebral angiography	156 (2.0%)	277 (3.6%)	769 (9.9%)	<0.0001	<0.0001
Electrocardiogram	1,739 (22.4%)	2,396 (30.8%)	2,525 (32.5%)	<0.0001	0.0119
None of the previous	5,465 (70.3%)	4,744 (61.0%)	3,863 (49.7%)	<0.0001	<0.0001
Laboratory tests					
Total cholesterol	3,622 (46.6%)	3,827 (49.2%)	3,698 (47.6%)	0.0007	0.0149
Glycosylated haemoglobin, Type A1C	1,070 (13.8%)	1,339 (17.2%)	1,434 (18.4%)	<0.0001	0.0024
None of the previous	3,958 (50.9%)	3,720 (47.8%)	3,775 (48.5%)	<0.0001	0.1947
None of the previous drugs, diagnostic procedures, nor laboratory tests	1,652 (21.2%)	1,104 (14.2%)	388 (5.0%)	<0.0001	<0.0001

**Fig. 1:**
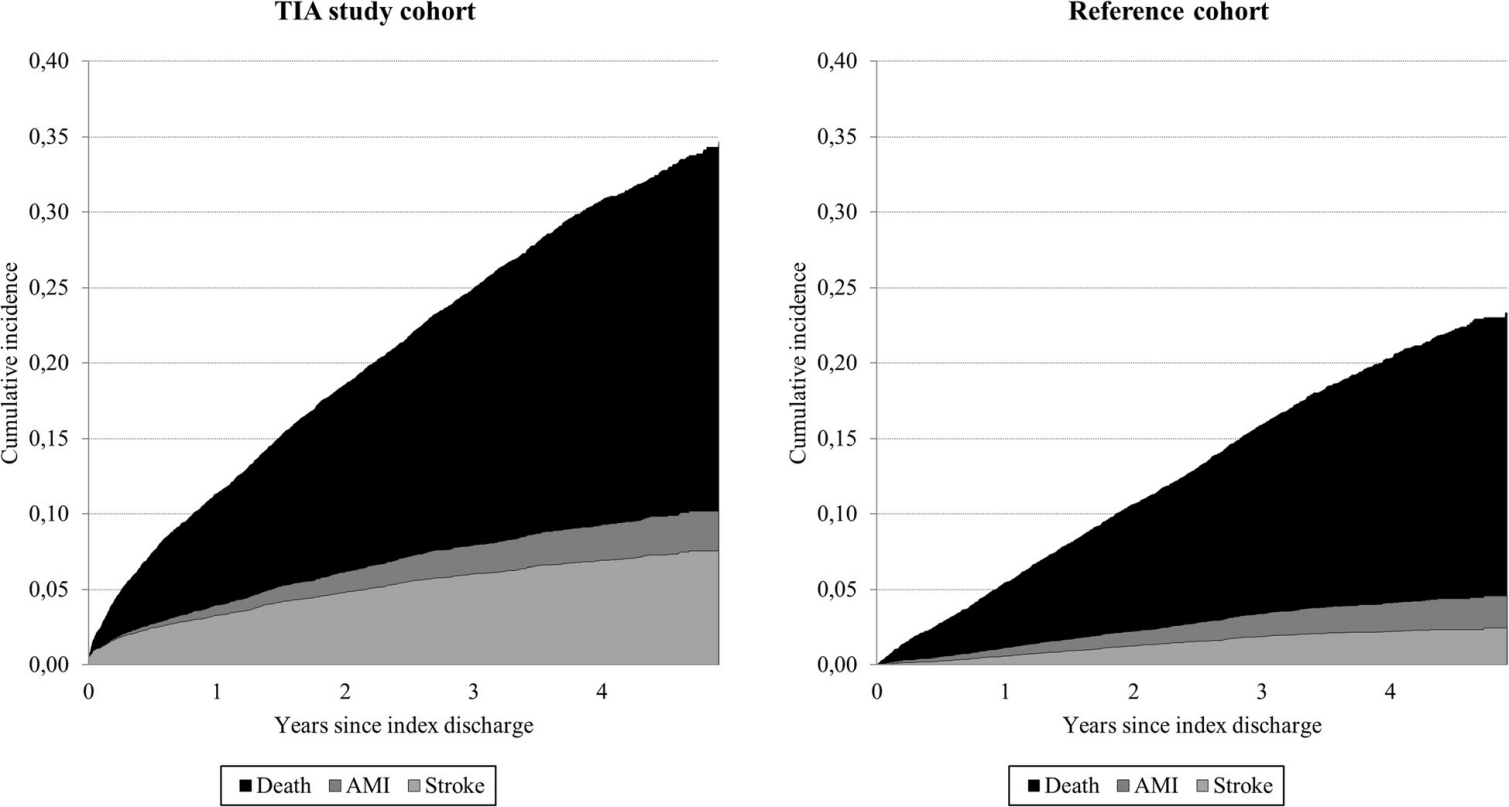
Cumulative mortality and hospital readmission for stroke and acute myocardial infarction (AMI) among study and reference cohort members

During the observation period, the study and reference cohort members accumulated 25,306 and 25,725 person-years of follow-up (in average 3.3 and 3.5 years per patient respectively) and generated 2,353 and 1,557 outcomes. Cumulative incidences are shown in Fig. 1. Among the study cohort members, cumulative mortality and hospital readmission for stroke or AMI were respectively 7.4, 3.3 and 0.7% after 1 year, and 24.5, 7.6 and 2.6% after 5 years from the index discharge. Among the reference cohort members, the corresponding figures were 4.3, 0.6 and 0.6% after 1 year, and 18.8, 2.4 and 2.1% after 5 years from the index discharge. The cumulative composite incidence rate was 2.1-fold and 1.5-fold higher among the study cohort than the reference cohort within 1 year and 5 years after the index discharge, respectively.

**Fig. 2:**
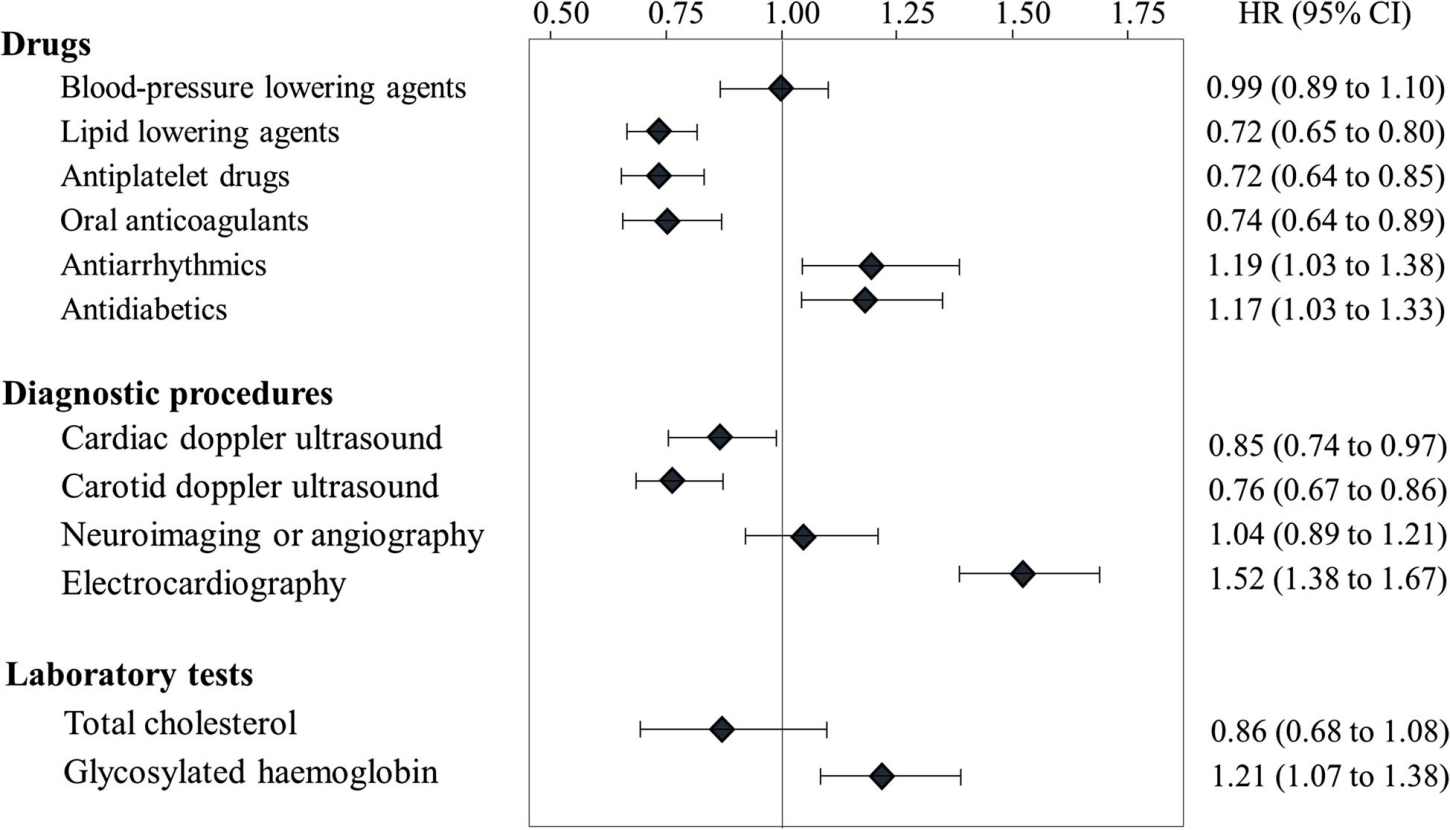
Hazard ratio (and 95% confidence interval) for the association between healthcare exposure and time of the composite outcome onset

Figure 2 shows that TIA cohort members who experienced exposure to selected drugs (lipid-lowering agents, antiplatelet and oral anticoagulants) and diagnostic procedures (i.e., carotid and cardiac doppler ultrasonography) during follow-up had significant lower risk of the composite outcome than those who did not use these services. Conversely, patients who took antidiabetics and antiarrhythmics, those who underwent electrocardiography, and those tested for glycosylated haemoglobin, type A1C levels (HbA1C), were at higher risk.

**Fig. 3:**
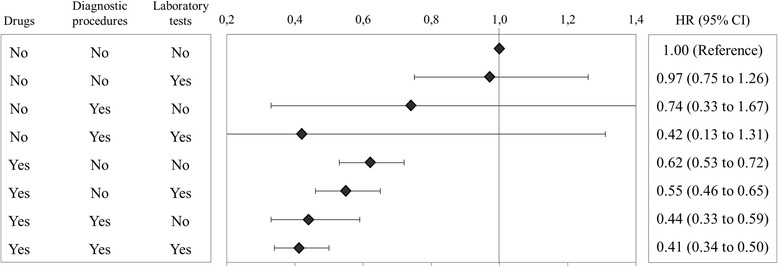
Joint action of exposure to whichever drug, diagnostic procedure and laboratory test on the risk of the composite outcome

The joint action of exposure to whichever drug, diagnostic procedure and laboratory test on the risk of the composite outcome is shown in Fig. 3. Patients who during follow-up used at least a drug, a diagnostic procedure and a laboratory test had a 59% reduced risk (95% CI, 50 to 66%) with respect to those who did not use any of these services. Drug therapies exerted higher protective action than other services since patients who took drugs, but neither used diagnostic procedure nor laboratory tests, had a significant reduced risk of 38% (95% CI, 28 to 47%).

The risk reduction was confirmed by including the 6,063 patients who during the index hospital admission were underwent to neuroimaging, having exposed to medical care of this subgroup a 54% reduced risk (95% CI, 42 to 64%) with respect to those who did not use any medical services.

## Discussion

Our study shows that: (i) management of TIA in daily clinical practice of the Italian Lombardy Region is suboptimal since in the first year after discharge little less than one in ten patients (8.6%) did not use blood pressure- and lipid-lowering agents, antidiabetics, antiplatelet and oral anticoagulants, nearly half of them (49.7%) have not been underwent to diagnostic procedures such as carotid and cardiac doppler ultrasonography, while one in twenty of them (5.0%) did not show any evidence of exposure to drugs, diagnostic procedures nor laboratory tests; (ii) patients with TIA diagnosed in hospital represent a high-risk group since 11.4 and 34.7% of them died or was hospitalized for stroke or AMI within 1 year and 5 years from index discharge respectively; (iii) the onset of a TIA represents a special opportunity for preventive intervention, since patients who used drug therapies and diagnostic procedures commonly recommended by guidelines showed a 59% risk reduction of death or cardiovascular hospitalization with respect to those who did not use any medical device.

A growing body of literature confirms the high rates of death and re-hospitalization during the first year after TIA. Patients recruited into clinical trials after a TIA have an annual risk of important vascular events (death from all vascular causes, non-fatal stroke, or non-fatal myocardial infarction) between 4 and 11% [22–24]. Similar figures have been reported from population-based observational studies being the corresponding rate around 9% per year [25], while the 10-year risk of stroke, myocardial infarction, or vascular death was reported to be 43% [5]. Our study supports the available data showing that patients discharged with a diagnosis of TIA had from 1.5-fold to 2.1-fold higher risk of unfavourable outcomes with respect to individuals of the same age and gender with no signs of cerebrovascular event.

Because medicines, diagnostic and screening investigations play an important role in secondary prevention for TIA patients [26–29], changes to the usual medicine regimen and diagnostic procedures can be expected for them [30]. Consistently, our study showed that users of blood pressure- and lipid-lowering agents, antiplatelet therapy, cardiac and carotid doppler ultrasonography and screening for diabetes and hyperlipidaemia increased after TIA occurrence. However, similarly to other reports [31–33], our findings show that several patients did not receive even one of these drugs and diagnostic procedures within one year after discharge.

Other findings are noteworthy and also warrant discussion. First, adverse outcomes were reduced of more than a 25% among patients who were treated with lipid-lowering, antiplatelet and anticoagulant agents. Although this is an expected finding according to current evidence-based recommendations on the prevention of future stroke among survivors of ischemic stroke or TIA [34], we suspect that the observed protective effect might be in part explained by the fact that drug exposures likely reflect general medical attention. This would explain because a similar protective effect was conferred by so different therapeutic actions. Second, patients who during follow-up used antidiabetic agents, as well as those who underwent a screening with glycosylated haemoglobin (HbA1C), were at higher risk for the considered outcomes likely because diabetes is known to be associated with unfavourable consequences after a cerebrovascular event occurs [35]. Analogously, patients who during follow-up used antiarrhythmic agents, and those who underwent electrocardiography, had worse prognosis likely because these exposures are proxies of high cardiac risk. Third, we observed that during the one-year period before the TIA occurrence, patients at their first episode of TIA (cases) had higher use of drugs for treating hypertension, cardiac arrhythmias and type 2 diabetes mellitus than patients who did not show evidence of cerebrovascular outcomes (controls). This finding supports the important role of hypertension, arrhythmias and diabetes as risk factors for TIA [36–38], confirming the potential benefits of primary prevention through the control of these conditions.

The present study has several elements of strength. First, the investigation was based on a very large unselected population, which was made possible because in Italy a cost-free healthcare system involves virtually all citizens. Second, the drug prescription database provided highly accurate data, because pharmacists are required to report prescriptions in detail in order to obtain reimbursement, and incorrect reports about the dispensed drugs have legal consequences [39]. Third, patients were included if there was no previous evidence of cerebrovascular disease over several years, which makes our results relevant in the setting of primary prevention of stroke. Finally, patients were identified from the point of the first episode of hospitalization for TIA and the complete sequence of following healthcare services supplied by the NHS was known.

Our study has also some limitations. One, because of privacy regulations, hospital records were not available for scrutiny, which means that diagnosis of TIA could not be checked. Owing the lack of evidence from up-to-date studies performed in the healthcare system of the Region we study, misclassification cannot be completely excluded in our setting. For example, if benign conditions (e.g., migraine, syncope and seizure) were mistaken for TIA [40], a portion of patients at low risk had been included in our cohort. Although our main result was confirmed by including patients underwent to neuroimaging at the index hospitalization, the lack of clinical information useful for risk stratification and prognostication, is a main cause of systematic uncertainty of our study.

Two, not all patients who experience a TIA are hospitalized [41], thus implying that our study selectively included more severe patients, and/or those at whom more clinical attention was reserved. Three, drug treatment was derived from drug prescriptions, which requires, however, the assumption that drug prescription corresponds to drug consumption [42]. There is, however, no guarantee that this is always the case, and indeed it is likely that in a number of patients the prescribed drugs are not consumed. This implies, however, that in the real-world, the actual use of medicines may be even still less than the measured prevalence of drug users.

Finally, the more relevant question in interpreting our findings is whether the observed results are due to our inability to fully account for lower adherence with guidelines to those patients at higher risk of adverse outcomes. That is, the reduction in adverse outcome associated with a better adherence to guidelines and a higher use of healthcare services might rather have been generated by a worse clinical profile, e.g., the severity of ischemic lesion and comorbidities. Yet, TIA clinical presentation (i.e., anterior circulation/posterior circulation TIA) could affect the adherence to medicaments, since, as well known, posterior circulation TIA are often misdiagnosed, representing a challenge in vascular neurology [43, 44]. However, because it is well known that patients with worse clinical profile more likely adhere to medical care [45], the observed risk reduction might be larger than that quantified by our study. Thus, our results are likely to reflect the favourable effect offered by healthcare services based on guidelines on cardiovascular protection.

## Conclusion

In summary, our findings suggest the need for intensive medical attention after TIA occurrence in an effort to prevent disabling stroke, other cardiovascular accidents and death. Patient outcomes may be improved by efforts aimed at increasing both patient and provider adherence to evidence-based guidelines.
